# Improving risk prediction for target subpopulations: Predicting suicidal behaviors among multiple sclerosis patients

**DOI:** 10.1371/journal.pone.0277483

**Published:** 2023-02-16

**Authors:** Yuval Barak-Corren, Victor M. Castro, Solomon Javitt, Matthew K. Nock, Jordan W. Smoller, Ben Y. Reis

**Affiliations:** 1 Predictive Medicine Group, Boston Children’s Hospital Informatics Program, Boston, MA, United States of America; 2 Technion, Israeli Institute of Technology, Haifa, Israel; 3 Partners Research Information Systems and Computing, Boston, MA, United States of America; 4 Department of Psychiatry, Massachusetts General Hospital, Boston, MA, United States of America; 5 Department of Psychology, Harvard University, Boston, MA, United States of America; 6 Psychiatric and Neurodevelopmental Genetics Unit, Center for Human Genetic Research, Massachusetts General Hospital, Boston, MA, United States of America; 7 Harvard Medical School, Boston, MA, United States of America; Universita degli Studi di Napoli Federico II, ITALY

## Abstract

Several recent studies have applied machine learning techniques to develop risk algorithms that predict subsequent suicidal behavior based on electronic health record data. In this study we used a retrospective cohort study design to test whether developing more tailored predictive models—within specific subpopulations of patients—would improve predictive accuracy. A retrospective cohort of 15,117 patients diagnosed with multiple sclerosis (MS), a diagnosis associated with increased risk of suicidal behavior, was used. The cohort was randomly divided into equal sized training and validation sets. Overall, suicidal behavior was identified among 191 (1.3%) of the patients with MS. A Naïve Bayes Classifier model was trained on the training set to predict future suicidal behavior. With 90% specificity, the model detected 37% of subjects who later demonstrated suicidal behavior, on average 4.6 years before the first suicide attempt. The performance of a model trained only on MS patients was better at predicting suicide in MS patients than that a model trained on a general patient sample of a similar size (AUC of 0.77 vs. 0.66). Unique risk factors for suicidal behavior among patients with MS included pain-related codes, gastroenteritis and colitis, and history of smoking. Future studies are needed to further test the value of developing population-specific risk models.

## Introduction

Suicide is one of the leading causes of death worldwide, with a death rate that has not decreased and even increased over the past 100 years [1, 2]. A major obstacle to preventing suicide has been the inability to accurately identify those at risk for suicidal behavior. A recent meta-analysis of studies focused on predicting suicidal behavior over the past 50 years found that most studies attempted to predict suicidal behavior outcomes using only one variable at a time, with each variable associated with only a small increase in risk [3].

Recent advances such as the development and implementation of electronic health records (EHRs) and machine learning methods have enabled researchers to combine information about numerous risk factors, resulting in much greater predictive accuracy [4–6]. Prior studies using these methods have focused on the development of models that can be used to predict suicidal behavior in the overall population of patients present in a given healthcare system.

From the perspective of precision medicine, generating predictive models for specific subgroups of patients may yield more accurate predictions and identify risk factors that are especially important among that subgroup [7]. Here we tested this assumption by developing a predictive model of risk of suicidal behavior among patients with multiple sclerosis (MS), a subpopulation of patients shown in prior studies to be at elevated risk of suicidal behavior, with a risk of suicide attempt and death that is approximately two times higher than the general population [8–11]. We hypothesized that the predictive model would include well-known risk factors for suicidal behavior in the general population (e.g., depression, substance use disorders) as well as risk factors for suicidal behavior observed in prior studies of patients with MS (e.g., physical disability) [12].

## Methods

### Sample and data collection

A retrospective cohort design was used. We analyzed data from the Partners Healthcare Research Patient Data Registry (RPDR), an EHR data warehouse covering 4.6 million patients from two large academic medical centers in Boston: Massachusetts General Hospital (MGH) and Brigham and Women’s Hospital (BWH), as well as other community and specialty hospitals in the Boston area, including McLean Hospital. All outpatient and inpatient hospital visits between 1998 and 2014, inclusive, at Partners Healthcare were included.

Within the RPDR, 15,117 patients had a diagnosis of Multiple Sclerosis (ICD-9 of 340) and met our three inclusion criteria of: (1) three or more visits, (2) 30 days or more between first and last visits, and (3) at least one healthcare encounter recorded when the patient’s age was between 10 and 90 years. We collected all demographic, diagnostic, procedure, laboratory, and medication data for each visit. For the case cohort, all data recorded after the first instance of suicidal behavior were excluded.

### Suicidal behavior case definition

The outcome variable of suicidal behavior was determined based on an ICD-9 case-definition developed by this group reported in an earlier paper and included both non-lethal and lethal suicide attempts [4]. Over 2,700 notes for 520 individuals were reviewed to establish codes that best identified suicide attempt cases, generating the following list of ICD-9 codes: E95*, 965.*, 967.*, 969.*, 881.*. These were supplemented by obtaining death certificates from the Commonwealth of Massachusetts, to capture completed suicides not recorded in the RPDR. A total of 852 death certificates between 1997 and 2010 with a “manner of death” of suicide (ICD-9: E95* or ICD-10 X60-X84, Y87.0) also were included as case patients.

### Model development

The cohort was randomly divided into two equally sized sub-cohorts of training and testing (validation) sets. The training set was used to develop the model and the testing set was used for model validation and evaluation of its performance. As in previous studies [4, 13] we used a Naïve Bayesian Classifier (NBC) model to estimate a patient’s risk for suicidal behavior. This modelling approach has two main strengths: (a) NBC is a very lean and efficient algorithm, thus making model development highly scalable for handling many independent variables, and (b) the coefficients of the resulting model are derived from the log of the odds-ratio and are thus very straightforward to interpret, unlike other “black-box” modeling methods (models that do not disclose the factors driving the model). Models were developed using R version 3.1.1, model performance was evaluated using the pROC package, and ggplot2 was used for plots generation. A more detailed description of model development is found in our previous publications [4, 13].

The models included data on demographics, diagnostic codes, lab results (with indication of normal/low/high values), and prescribed medications (true/false values without dosage). Data were collected up to but not including the first suicidal event for the cases, and for all observed time periods for the controls. For each independent input variable in the training dataset (e.g. diagnoses, medications, etc.), we assigned a partial risk score based on the ratio of its prevalence among cases vs. controls. The score was calculated on a logarithmic scale, where negative scores represented “protective” factors (negatively associated with suicidal behavior) and positive scores represented “adverse” risk factors (positively associated with suicidal behavior).


$$PRS_{i}=log[\left(Cases\;with\;i/Cases\;without\;i)/(Controls\;with\;i/Controls\;without\;i\right)]$$

Partial Risk Score (PRS) for variable i (e.g. use of specific medication, a lab test, or a certain diagnosis) was calculated using the log of the odds ratio for that variable.

The model generates a continuous score that is the sum of all partial risk scores. In preparation for model validation, these continuous results were transformed into a binary outcome of yes/no subject had suicidal behavior based on selecting risk score thresholds using the training set. Thresholds were selected to achieve benchmark specificities of 90% and 95% (ie, choosing the 90% and 95% percentiles of the training controls’ scores as the cutoff score).

### Model validation

We validated the model on the testing set using a simulated prospective approach: for each patient we ordered all available variables (diagnoses, medications, lab tests), from oldest to newest, and calculated the cumulative score at each time point using the sum of the partial risk scores associated with each code or variable. Each patient’s overall risk-score at each time point was calculated by combining the partial risk scores preceding that time point. The patient’s score was interpreted using the maximal risk score obtained over time and compared to the thresholds selected during the training phase to achieve 90% and 95% specificities, respectively, and the sensitivity and timeliness of prediction at these levels of specificity were measured.


$$Risk_{t}=PRS_{i}+PRS_{j}+PRS_{k}+\ldots .+PRS_{z}$$

$$ORS=max(Risk_{t0},Risk_{t1},Risk_{t2},\ldots .Risk_{t-n})$$

The total risk score at time point t is calculated as the sum of all partial risk scores (PRSs) up to that time point (PRS i, j, k, …, z all occurred before time point t). The overall risk score (ORS) for each subject, is calculated using the maximal value of all total-risk-scores calculated.

### Ethics

The study was approved by Partners Healthcare Institutional Review Board. The need for consent was waived by the ethics committee. All data were fully anonymized before the start of the study, identifiers were removed and dates were offsetted.

## Results

### Model composition

Among the total 15,117 patients with a diagnosis of Multiple Sclerosis (MS), we identified 197 (1.3%) cases with suicidal behavior. All other patients were considered controls. On average, each subject was followed for a period of 8.4 years. Cases had a mean follow-up of 11.2 years (95% CI 10.4–11.9) and controls had a mean follow-up of 8.3 years (95% CI 8.2–8.4). Overall, the analysis included a total of 61,300 person-years. The median follow-up time available before the first ICD-9 code of Multiple Sclerosis was 3.0 years. The MS diagnosis preceded the suicidal event in 61% of the cases (*n* = 120), whereas the opposite was the case in 32% (*n* = 64), and both events were recorded on the same date at 7% of the cases (*n* = 13). We excluded 91 cases from our analysis due to lack of data: 27 cases without any documentation prior to the first suicidal event and 64 cases where suicide preceded the index MS diagnosis. This yielded a final set of 106 cases (0.7%) and 14,920 controls (99.3%). For included cases, index MS diagnosis preceded the suicidal event by an average of 5.2 years (21 days to 15.6 years, 95% CI of 4.35–6.05).

Table 1 summarizes the demographic characteristics of all patients recorded within the RPDR data warehouse (not including the excluded cases), as well as a comparison of these demographic features between cases with suicidal behavior and controls without such behavior. As seen in the table, the female to male ratio in our MS cohort was 2.7:1 (73% of subjects were women and 27% men). The mean age at the end of our study time period (12/31/2015) was 54.5 years among the MS patients, slightly older than the average age of 52.6 years in the entire RPDR dataset (*p* < 0.001). The MS cohort had a greater proportion of white people (80.1%) and a lower proportion of Hispanic people (2.6%) compared to the baseline rate in the RPDR dataset (72.7% and 6.9% respectively). Overall, patients with MS were more likely to be married than single in comparison to the general RPDR population (55.9% married and 26.6% single vs. 47.7% married and 35.2% single), though those with suicidal behavior were more likely to be single than married (34.9% married and 42.5% single, χ^2^<0.001).

**Table 1:** Demographic features of study population, including the sub cohorts of suicide cases and controls.

Feature	All Patients (n = 1,931,672)	All MS Patients (n = 15,117)	Controls (n = 14,920)	Cases (n = 197)	P-val (cases)
**Gender**					
Men	41.9% (n = 809276)	26.7% (n = 4043)	26.7% (n = 3979)	32.5% (n = 64)	0.08
Women	58.1% (n = 1122221)	73.2% (n = 11072)	73.3% (n = 10939)	67.5% (n = 133)	0.081
**Age**	52.6 [52.6–52.6]	54.6 [54.3–54.8]	54.5 [54.3–54.8]	56.7 [54.4–59]	0.067
**Ethnicity**					
Asian	3.7% (n = 71470)	1.1% (n = 170)	1.1% (n = 168)	1% (n = 2)	~1
Black	6.4% (n = 123903)	5% (n = 757)	5% (n = 746)	5.6% (n = 11)	0.835
Hispanic	6.9% (n = 133411)	2.6% (n = 399)	2.6% (n = 389)	5.1% (n = 10)	0.054
Other	10.3% (n = 198189)	11.2% (n = 1686)	11.2% (n = 1671)	7.6% (n = 15)	0.14
White	72.7% (n = 1404699)	80.1% (n = 12105)	80.1% (n = 11946)	80.7% (n = 159)	0.893
**Marital Status**					
Divorced	5.1% (n = 97781)	7.8% (n = 1183)	7.8% (n = 1163)	10.2% (n = 20)	0.276
Married	47.7% (n = 922075)	55.8% (n = 8441)	56.1% (n = 8368)	37.1% (n = 73)	<0.001
Other	6.1% (n = 118081)	4.4% (n = 667)	4.4% (n = 662)	2.5% (n = 5)	0.265
Partner	0.2% (n = 2991)	0.2% (n = 36)	0.2% (n = 35)	0.5% (n = 1)	~0.964
Separated	1.1% (n = 20510)	1.2% (n = 186)	1.2% (n = 184)	1% (n = 2)	~1
Single	35.1% (n = 678704)	26.7% (n = 4034)	26.5% (n = 3949)	43.1% (n = 85)	<0.001
Widowed	4.7% (n = 91530)	3.8% (n = 570)	3.7% (n = 559)	5.6% (n = 11)	0.247
**% Cases**	1.3% (n = 25730)	1.3% (n = 197)	0% (n = 0)	100% (n = 197)	

Age is the age taken at 12/31/2015.

**Fig 1:**
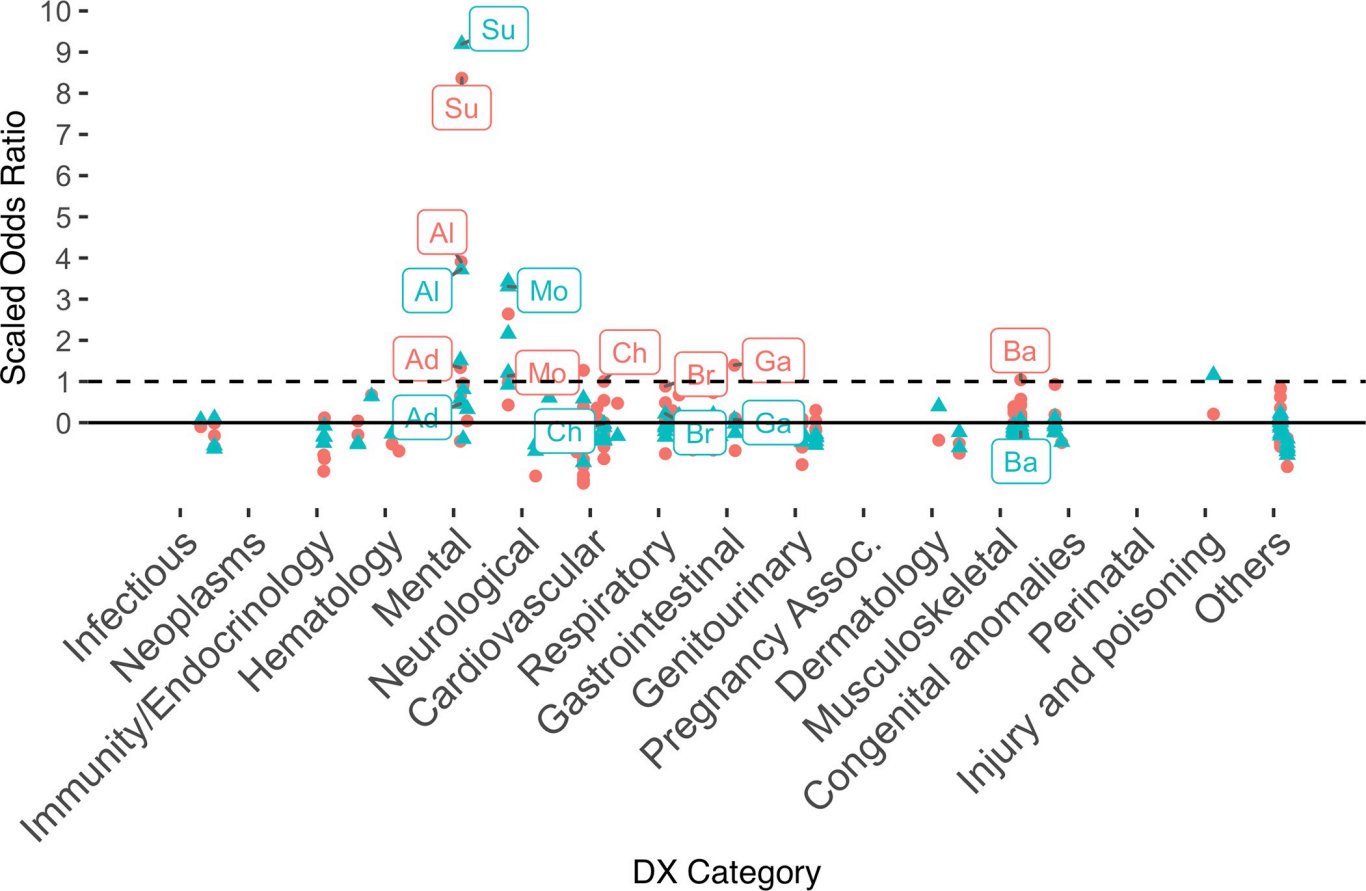
Manhattan plot comparing risk factors for suicidal behavior among MS patients (blue) vs. the general population (red). Values are shown as odds-ratios, normalized within each group—e.g. Substance abuse [Su] has an odds ratio of 13.93 among MS patients and 10.66 among the general population, which were scaled to 8.17 SD from the mean for MS and 8.90 SD from the mean to the general population. Key: Al—alcohol-related disorder, Su—substance-related disorder, Ad—adjustment disorder, Mo—mood disorders, Ch—chronic obstructive pulmonary disease, Br—Brachial neuritis or radiculitis NOS, Ga—gastroenteritis, Ba—back problems.

In addition to the demographic differences outlined above, there were also significant clinical differences between cases and controls (Table 2). These findings include both risk factors relevant for the entire population MS and non-MS patients alike, as seen in a previous study conducted using the same dataset (Barak-Corren et al., 2017), and novel risk factors that are specific to the MS cohort. Examples for risk-factors associated both with the general population as well as with the MS-cohort include: “Drug abuse, unspecified use” (OR = 13.9), “Alcohol abuse, unspecified drinking behavior” (OR = 7.9), and mental-health diagnoses like “Neurotic depression” (OR = 6.2). Risk factors unique to the MS cohort included: “chest pain” with an OR of 3.9 among MS patients vs. 1.2 in the general population (could be associated with the “MS hug”–a girdling pain around the torso as a result of dysesthesia due to MS) [14], “Other and unspecified noninfectious gastroenteritis and colitis” (OR = 4.5 vs. 1.3), and a “history of tobacco use” (OR = 3.9 vs. 0.9). Table 2 and Fig 1 compare the odds ratio of different diagnostic codes, grouped according to the Clinical Classification Software (CCS), between the MS cohort and the general population. The odds ratios are scaled within each cohort so that each value is represented as the number of standard deviations from the mean. This is done to correct for the overall higher ratios in the MS cohort that are associated with the great difference in sizes between these cohorts (*n* = 15K in the MS cohort vs. *n* = 1.9M in the general population cohort).

**Table 2:** Comparison of odds-ratio of different diagnostic codes among MS cohort vs. the general population.

	MS Cohort			Entire Cohort			
Diagnosis	Controls	Cases	OR	Controls	Cases	OR	P-Value
Other, mixed, or unspecified drug abuse, unspecified use	123	11	13.9 (8.18)	5,483	684	10.7 (8.9)	0.009
Alcohol abuse, unspecified drinking behavior	216	11	7.9 (3.81)	15,458	916	5.1 (3.59)	0.109
Neurotic depression	642	23	6.2 (2.57)	19,563	572	2.5 (1.16)	<0.001
Other and unspecified noninfectious gastroenteritis and colitis	737	20	4.5 (1.35)	31,622	493	1.3 (0.05)	<0.001
Adjustment reaction with brief depressive reaction	383	11	4.4 (1.3)	10,906	220	1.7 (0.43)	<0.001
Other chronic pain	634	17	4.3 (1.23)	10,501	228	1.9 (0.55)	<0.001
Depressive disorder, not elsewhere classified	2,645	50	4.1 (1.11)	80,128	2,061	2.2 (0.87)	<0.001
Major depressive disorder, single episode, unspecified degree	448	12	4.1 (1.1)	12,649	687	4.6 (3.19)	0.002
Brachial neuritis or radiculitis NOS	636	16	4 (1)	13,363	160	1 (-0.24)	<0.001
Other chest pain	1,302	29	3.9 (0.97)	51,493	740	1.2 (-0.05)	<0.001
Unspecified affective psychosis	473	12	3.9 (0.94)	14,284	796	4.8 (3.31)	0.012
History of tobacco use	895	21	3.9 (0.92)	40,337	402	0.9 (-0.4)	<0.001
Pain in limb	3,784	60	3.8 (0.89)	148,742	2,030	1.2 (-0.1)	<0.001
Tobacco use disorder	1,228	27	3.8 (0.87)	51,984	1,271	2.1 (0.77)	<0.001
Acute bronchitis	717	17	3.8 (0.85)	33,946	588	1.5 (0.19)	<0.001
“Protective Factors” (OR < 1)							
Demyelinating disease of central nervous system, unspecified	4,260	27	0.9 (-1.26)	2,936	25	0.7 (-0.52)	<0.001
Unspecified disease of spinal cord	2,815	17	0.8 (-1.28)	6,088	51	0.7 (-0.53)	<0.001
Other nonspecific abnormal results of function study of brain and central nervous system	2,127	11	0.7 (-1.38)	4,278	40	0.8 (-0.45)	<0.001
Other conditions of brain	1,609	7	0.6 (-1.46)	5,574	18	0.3 (-0.95)	<0.001

Odds ratios are shown alongside their distance from the mean in standard-deviations (Z-score).

### Model performance

**Fig 2:**
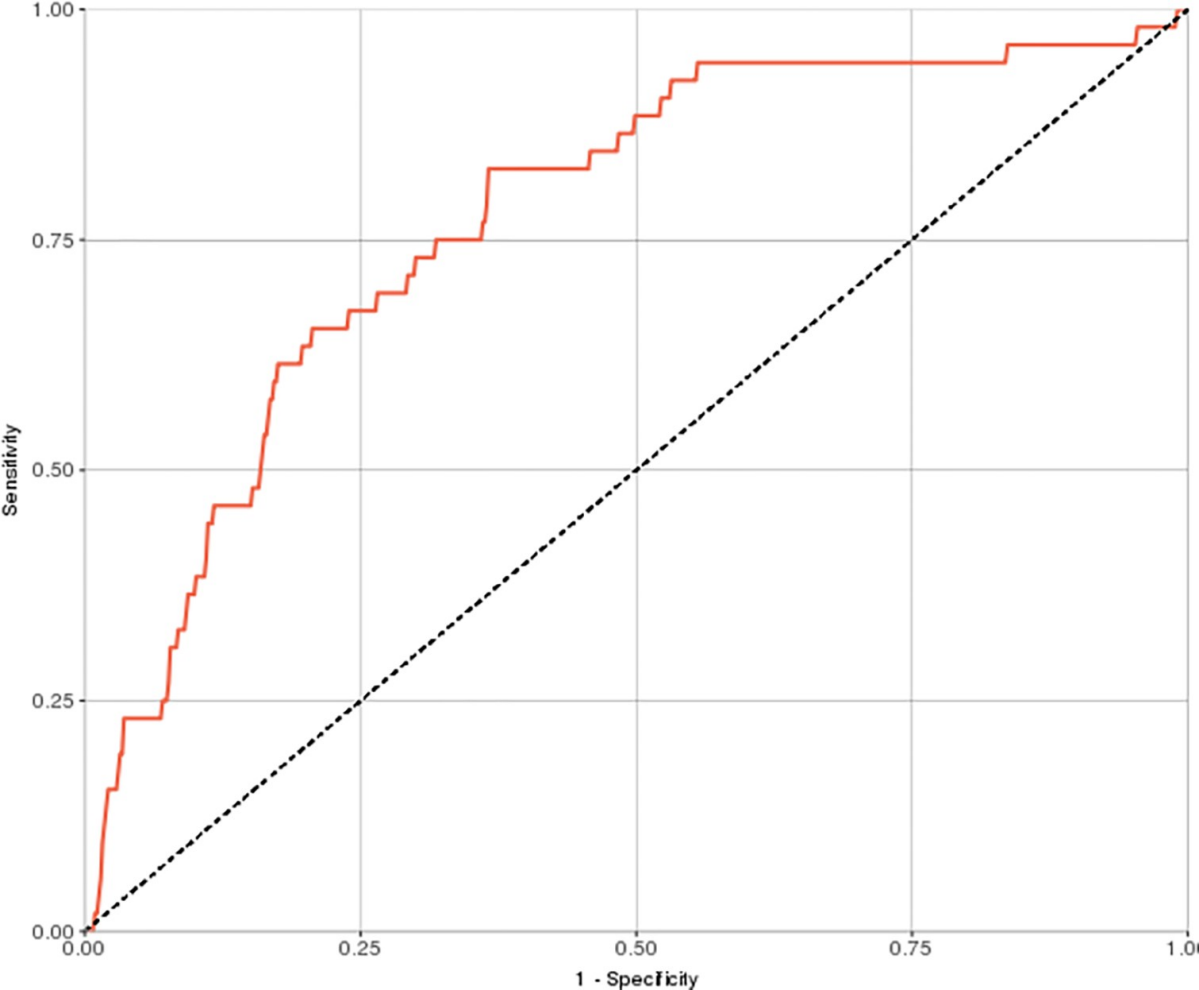
Model ROC curve (AUC = 0.77). Overall model performance is shown as a tradeoff between the sensitivity of the model (y-axis) and specificity (x-axis). The dotted line represents chance prediction and the top left point in the curve is the point of maximal sensitivity and specificity.

Relying only on coded information commonly available in the EHR, the model successfully predicted suicidal behavior with an overall area under the ROC curve of 0.77 (Fig 2). With 90% specificity, the model detected 37% of the subjects who later demonstrated suicidal behavior. Consistent with the low base-rate of suicidal behavior in the full cohort, the PPV was 2% compared with the 0.7% baseline prevalence in the included cohort, i.e. 2.86 times higher (Table 3).

**Table 3:** Summary statistics and overall performance of the suicide prediction model.

Spec	Sens	PPV	NPV	TP	TN	FP	FN	LR+	LR-
99%	2%	1%	99%	1	7433	75	51	2.00	0.99
95%	23%	3%	99%	12	7133	375	40	4.60	0.81
90%	37%	2%	100%	19	6757	751	33	3.70	0.70
80%	63%	2%	100%	33	6006	1502	19	3.15	0.46
48%	90%	1%	100%	47	3594	3914	5	1.73	0.21

Spec = Specificity, Sens = Sensitivity, PPV = Positive Predictive Value, TP = True Positive, TN = True Negative, FP = False Positive, FN = False Negative, LR+/- Positive and negative likelihood ratio.

### Testing variability in model performance by time and sample size

We also examined the average time that the model was able to predict suicidal behavior in advance of an individual receiving a case-defining diagnosis for suicidal behavior. Setting specificity at 90%, the model identified 37% (*n* = 19) of the cases on average 4.6 years before the case-defining code was recorded in the EHR. Increasing the model specificity to 95% (i.e. only 5% false positives), our classifier correctly predicted suicide in 23% (*n* = 12) of the cases, on average 3.4 years prior to the actual event.

Finally, to account for the fact that our MS-specific model was trained on a much smaller set of data, we developed a similar Naive Bayes model on a training set of equal size to that of the MS sample from the general population, and then tested this model on the same validation set of individuals diagnosed with MS as the MS-specific model. The results were an AUC of only 0.66 (compared to 0.77 in the MS model). That being said, we found that the size of the cohort had a very large impact—when building the model using a training set of *n* = 864,000 subjects (50% of the general population) and applying it on the same MS validation set, we obtained an AUC of 0.81 (Table 4).

**Table 4:** Comparing predictive model performance on the MS validation set using different training sets to build the model.

	MS Population	General Cohort—Sampled	General Cohort—All
**Training Size**	N = 7,513	N = 7,513	N = 864,000
**Validation Size (MS Validation Set)**	N = 7,513	N = 7,513	N = 7,513
**Performance (AUC)**	77%	66%	81%

## Discussion

A growing body of research has attempted to leverage the large data resources of EHRs to predict risk of medically-important outcomes, including suicidal behavior [4–6]. The utility of such prediction models to specific at-risk subgroups may be compromised if subgroup-specific predictors are influential. Here, we examined the value of developing new predictive models for specific target populations, using the case of suicidal behavior among patients with MS, a group in whom prior studies have suggested elevated risk [8, 12]. Suicidal behavior was defined by ICD-9 codes related to suicide and self-inflicted poisoning/injury (E95*) as well as codes related to poisoning, open wounds of forearm/wrist, or firearms injuries (965*, 967*, 969*, 881*), in a case-definition that was validated on over 2,700 clinical notes. We found that deriving a subgroup-specific prediction model yielded improved prediction accuracy compared to a model developed in the full health system population. Although many risk factors for suicidal behavior ranked highly in both the general and MS-specific models (e.g., alcohol and drug abuse, mental health diagnoses), the MS-specific model revealed several condition-specific risk factors. These included factors such as chronic pain and adjustment reaction with brief depressive reaction (ICD-9 309.0). These findings add to risk factors identified in prior studies of suicide among those with MS, which have largely focused on sociodemographic factors such as age, education, and income [9, 12, 15]. The approach used in the current study of looking at the entire EHR allowed us to test a much wider range of risk factors than examined in prior studies, and the resulting risk factors identified are largely unique but have strong face validity. For instance, learning that one has been diagnosed with MS could lead to a depressive reaction, and MS is a condition characterized by the experience of chronic pain, fatigue, depression, and sleep disturbances. All are factors that are associated with increased risk of suicidal behavior, with suicide serving as a potential means of escaping physical or psychological pain [16, 17]. Future studies should also use the way in which patients perceive the severity and disability of their MS symptoms as a risk score. While we did not have access to such information, this can be captured as patient rating of these symptoms.

Our predictive model performed well in identifying patients with MS who would subsequently engage in suicidal behavior, with a level of accuracy (AUC = .77) and PPV (1–3%) that is in line with results from other studies that have used machine learning applied to EHR to predict suicidal behavior [4, 5]. Some have raised questions about whether a PPV as low as 2% is clinically actionable for the prevention of suicidal behavior [18]. Notably, however, PPVs in this range are similar to those in other areas of medicine that have resulted in favorable treatment outcomes, such as in the treatment of breast cancer and cardiovascular disease [17, 18]. Given the relatively low cost of follow-up assessments to determine level of suicide risk, we expect the PPVs achieved here to be clinically meaningful and actionable.

Future studies should test whether prediction performance can be similarly improved for other conditions and subpopulations. For instance, it may be that prediction can be improved by generating separate models for: those with vs. without mental disorders, children/adolescents vs. adults, males vs. females, and so on. On balance, these groups can overlap and so methods are needed to control the complexity of the many different lines that can be drawn in the data. In addition, we observed in the current study that generating a predictive model in a subpopulation led to reduced sample size on which to train our model, which in turn limited predictive accuracy. Thus, the increase in accuracy for more specific models must be weighed against the penalty one incurs for building a model in a smaller sample. This trade-off must be weighed in each new analysis and the best solution may depend on whether one values discovery of new risk factors within that subpopulation vs. overall predictive accuracy of the model.

These findings must be viewed in light of several key limitations. First, the sample size in our MS-specific model was relatively small, and the observed results would benefit from replication in a larger sample. Second, although our MS-specific model had a stronger AUC than the more general model, there is still much room for improvement in this accuracy. This represents an important direction for future research. Third, the NBC approach used has the benefit of transparency, thus avoiding concerns with “black-box” algorithms [19]; however, it is a relatively simple approach that does not take into account potential interactions among putative risk factors that undoubtedly exist (e.g. diagnosis of depression and prescription of antidepressants). Finally, any suicidal event that occurred after 01/01/2016 was not captured in our study, thus some cases may have been misclassified as controls. Fourth, these findings were obtained using data from only one healthcare system. Although our prior work has shown that results of such models obtained in one healthcare setting can be achieved when this process is replicated in other healthcare settings around the country [20], the generality of the current results is unclear until such replication is done in the case of MS and suicide. These limitations notwithstanding, the current study demonstrates that value of developing population-specific risk models for suicidal behavior and reveals several novel risk factors for suicidal behavior among those diagnosed with MS.
